# Complete chloroplast genome of *Angelica hirsutiflora* Liu et al. 1961 (Apiaceae)

**DOI:** 10.1080/23802359.2024.2335992

**Published:** 2024-04-05

**Authors:** Chi-Chun Huang, Tsai-Wen Hsu, Kuo-Hsiang Hung, Wei-Kuang Wang

**Affiliations:** a Taiwan Biodiversity Research Institute, Jiji, Nantou, Taiwan; b Graduate Institute of Bioresources, Pingtung University of Science and Technology, Pingtung, Taiwan; c Department of Environmental Engineering and Science, Feng Chia University, Taichung, Taiwan

**Keywords:** Chloroplast genome, *angelica hirsutiflora*, apiaceae

## Abstract

*Angelica hirsutiflora* Liu et al.^1961^, is a perennial herb in the Apiaceae family that is endemic to Taiwan. In this study, the complete circular chloroplast genome of *A. hirsutiflora* was reconstructed and annotated using Illumina sequencing. The size of the chloroplast genome is 154,266 bp, consisting of two inverted repeats (IRs, 25,075 bp) separated by a large single-copy region (LSC, 86,569 bp) and a small single-copy region (SSC, 17,547 bp). The GC content of the chloroplast genome is 37.6%. There are 114 different genes in the chloroplast genome of *A. hirsutiflora*, including 80 protein-coding genes, 30 tRNA genes and four rRNA genes. A maximum-likelihood phylogenetic analysis showed that *A. hirsutiflora* forms a distinct clade, and separated from other species within the genus *Angelica*. This study provided insights into the evolutionary relationships among different species of *Angelica*.

## Introduction

**Figure 1.**
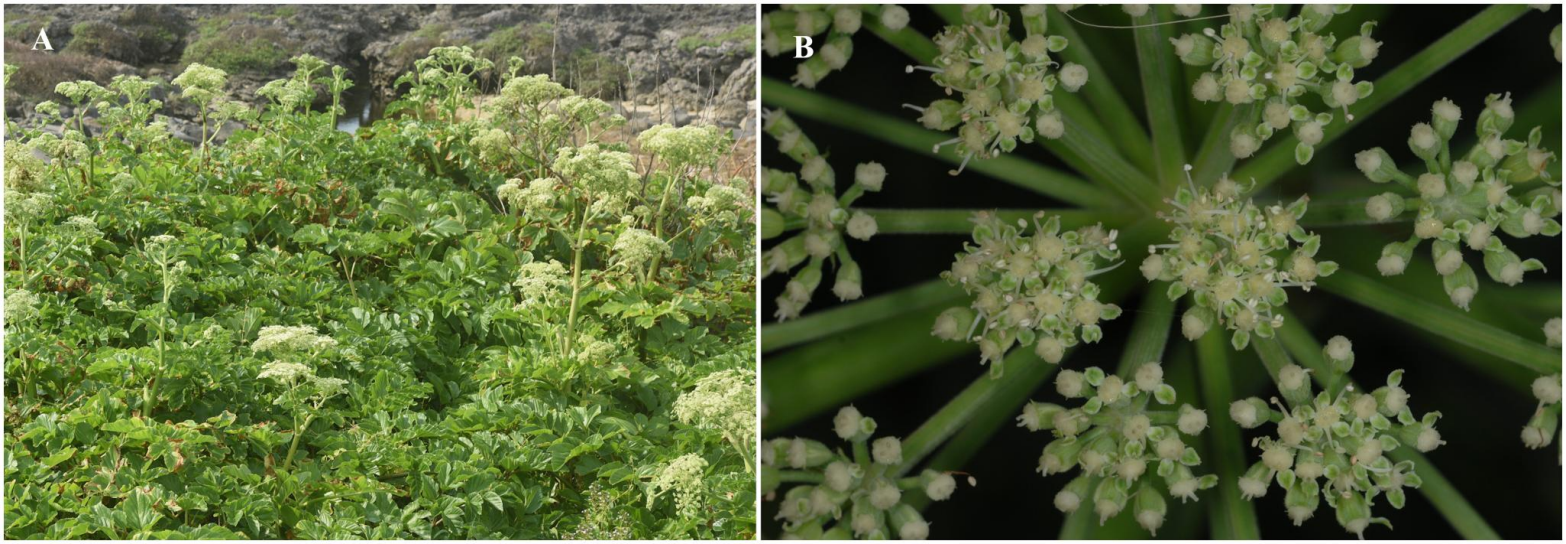
*Angelica hirsutiflora.* (A) Plant; (B) inflorescence. These images are photography by Tsai-Wen Hsu.

*Angelica hirsutiflora* Liu et al. 1961, is a perennial herb in the Apiaceae family that is endemic to Taiwan (Liu et al. 1961; Liu and Kao 1977; Kao 1993). It is mainly distributed in the coastal areas of northern Taiwan (Figure 1). *Angelica hirsutiflora* is considered an endangered species due to overexploitation and habitat destruction by humans (Editorial Committee of the Red List of Taiwan Plants 2017). *Angelica hirsutiflora* was formally descripted in Liu et al. (1961). Yamazaki (1990) treated *A. hirsutiflora* as a variety of *Angelica japonica* A. Gray, 1859, namely, *A. japonica* var. *hirsutiflora* (Liu, Chao & Chuang) T. Yamaz. 1990, which is distributed in the Ryukyu Islands and Taiwan. Seo et al. (2005) concluded that *A. hirsutiflora* can be taxonomically treated as independent species using allozyme analyses. According to Liao et al. (2022), *A. hirsutiflora* belongs to the littoral *Angelica* clade and is closely related to *A. japonica*. Therefore, we adhered the treatments of Kao (1993), Liu et al. (1961) and Liu and Kao (1977), which consider *A. hirsutiflora* a distinct taxonomic species. Recently, plastid phylogenomics has provided a tool to improve the reconstruction of phylogenetic relationships in *Angelica* L. (Wang et al. 2021). The chloroplast genome of *A. hirsutiflora*, obtained using next-generation sequencing, is characterized and assembled for the first time. This study enhances our understanding of the phylogenetic relationships among different species of *Angelica*.

## Materials and methods

### Plant collection

A wild individual of *A. hirsutiflora* was collected from Jinshan District (121°37’58”E, 25°15’36”N), New Taipei City, Taiwan. Despite being regarded as an endangered species, it is not a legally protected species in Taiwan. The collection location in this study is not a privately owned or protected area. No permits were required for this study. A specimen was deposited at the herbarium of the Endemic Species Research Institute (TAIE) (https://www.tesri.gov.tw/, Tsai-Wen Hsu, twhsu@tesri.gov.tw) under voucher number No. 24169.

### DNA extraction, sequencing, assembly, and annotation

Total genomic DNA was extracted from the leaf materials of *A. hirsutiflora* using CTAB extraction. The library was sequenced by the Illumina NovaSeq 6000 platform with the double terminal sequencing method (pair-end, 150 bp). Paired-end reads were assembled using GetOrganelle v. 1.7.7 (Jin et al. 2020). Annotation of the chloroplast genome was performed using GeSeq (Tillich et al. 2017). The gene graphical map of the chloroplast genome was constructed using CPGView (http://www.1kmpg.cn/cpgview) (Liu et al. 2023). The annotated genomic sequence has been deposited in GenBank under accession number OQ773546.

### Phylogenetic analysis

A phylogenetic tree was reconstructed with the complete chloroplast genomes of *A. hirsutiflora*, along with 37 samples, which represent 24other taxa in the Apiaceae family. The sequence alignment was conducted using the MAFFT online server (https://mafft.cbrc.jp/alignment/server/) (Katoh et al. 2019), and subsequently, a maximum likelihood phylogenetic tree was reconstructed by PhyML version 3.0 (http://www.atgc-montpellier.fr/phyml/) with GTR model (Guindon et al. 2010).

## Results

### Chloroplast genome features of A. hirsutiflora

**Figure 2.**
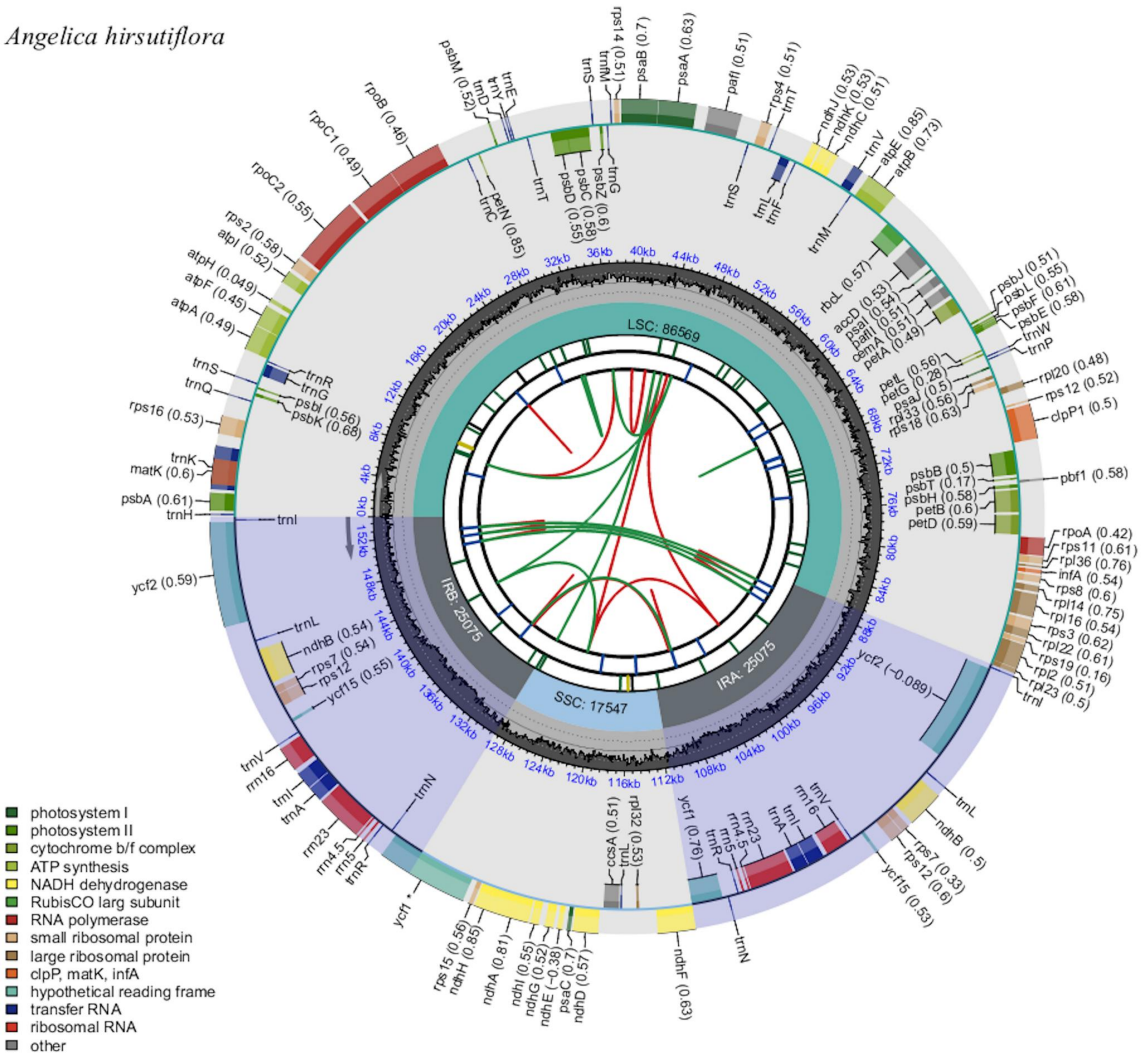
Schematic map of overall features of the chloroplast genome of *A. hirsutiflora.* Genes outside the circle are transcribed clockwise, while genes inside the circle are transcribed counterclockwise. Different functional groups are represented by different colors. The darker and lighter gray in the inner indicated the GC and at content, respectively.

A total of 14.7 Gb of clean data were generated. The structure of the chloroplast genome of *A. hirsutiflora* was circular, and the size was 154,266 bp, with an average depth of 441.79 x (Supplementary Figure 1). It was composed of a pair of inverted repeat regions (IRs: 25,075 bp) separated by a large single-copy (LSC) region of 86,569 bp and a small single-copy (SSC) region of 17,547 bp. The GC content of the chloroplast genome was 37.6%. There were 114 different genes in the chloroplast genome of *A. hirsutiflora*, including 80 protein-coding genes, 30 tRNA genes and four rRNA genes (Figure 2). Furthermore, 16 (ten protein-coding and six tRNA genes) genes contained one intron, and three protein-coding genes (*clpP1*, *pafI* and *rps12*) contained two introns. *rps12* has been recognized as a trans-splicing gene (Supplementary Figure 2).

### Phylogenetic analysis

**Figure 3.**
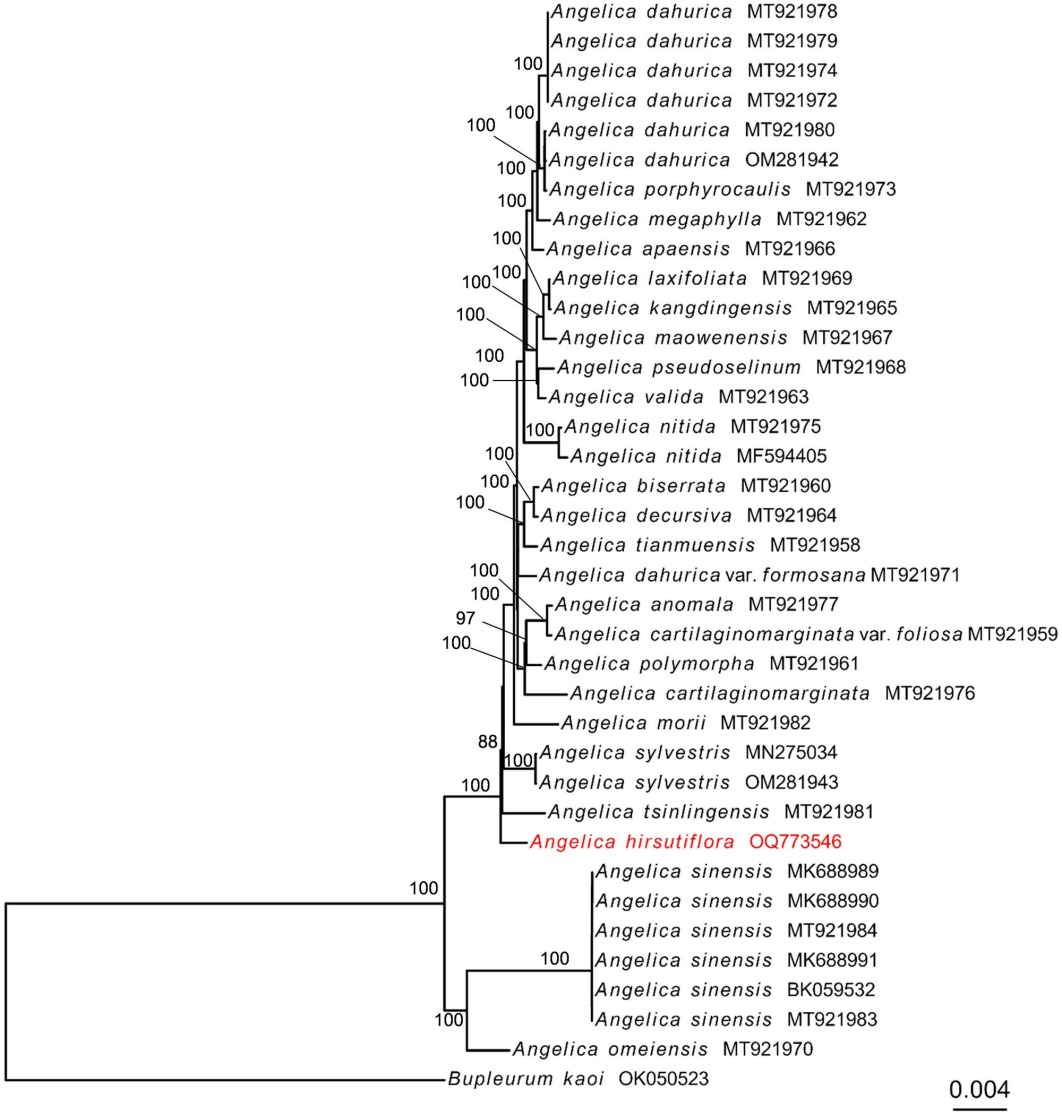
The phylogenetic tree was constructed using 38 chloroplast genomes sequences based on the maximum-likelihood analysis. The number on the branches are bootstraps values. *Angelica hirsutiflora* (OQ773546) obtained in this study are shown in red. The following sequences were used: *Angelica anomala* (MT921977)*, Angelica apaensis* (MT921966)*, Angelica biserrata* (MT921960)*, Angelica cartilaginomarginata* var. *foliosa* (MT921959)*, Angelica cartilaginomarginata* (MT921976), *Angelica dahurica* var. *formosana* (MT921971), *Angelica dahurica* (MT921972, MT921974, MT921978, MT921979, MT921980)*, Angelica decursiva* (MT921964)*, Angelica kangdingensis* (MT921965)*, Angelica laxifoliata* (MT921969)*, Angelica maowenensis* (MT921967)*, Angelica megaphylla* (MT921962)*, Angelica morii* (MT921982)*, Angelica nitida* (MT921975), *Angelica omeiensis* (MT921970), *Angelica polymorpha* (MT921961), *Angelica porphyrocaulis* (MT921973), *Angelica pseudoselinum* (MT921968), *Angelica sinensis* (MT921983, MT921984), *Angelica tianmuensis* (MT921958), *Angelica tsinlingensis* (MT921981), *Angelica valida* (MT921981) (Wang et al.^2021^), *Angelica dahurica* (OM281942)*, Angelica sylvestris* (OM281943) (Jiang et al.^2022^)*, Angelicasinensis* MK688989-MK688991 (Rong et al.^2019^), *Angelica sinensis* (BK059532) (Samigullin et al.^2022^), *Angelica nitida* (MF594405) (Deng et al.^2017^), *Angelica sylvestris* (MN275034) (Liao et al.^2019^) and *Bupleurum kaoi* OK050523 (Huang et al.^2022^).

The phylogenetic results showed that *Angelica* species formed a monophyletic clade, which was mainly divided into two groups (Figure 3). *Angelica hirsutiflora* formed an independent clade to other species of the genus *Angelica*. This study provides the chloroplast genome information of *A. hirsutiflora*, which can be used for species identification and phylogenetic analysis within *Angelica* species.

### Discussion and conclusion

Compared to other species of the littoral *Angelica* clade in Taiwan, *A. hirsutiflora* is the only species that is distributed in coastal areas, implying a possible distant relationship. The chloroplast genome of *A. hirsutiflora* was assembled and annotated for the first, and the genome was determined to be 154,266 bp in length, containing 114 genes. *Angelica hirsutiflora* formed a distinct clade, and separated from other species within the genus *Angelica* in the phylogenetic tree. Liao et al. (2022) and Wang et al. (2024) proposed that *A. hirsutiflora* is closely related to *A. japonica* according to nrITS sequences. *Angelica hirsutiflora* morphologically resembles *A. japonica* (Seo et al. 2005). However, the information of chloroplast genome of *A. japonica* is absent in the NCBI database. To better understand its phylogenetic relationships, additional chloroplast genomes from the littoral *Angelica* clade are urgently needed. This study has contributed to the enlargement of the chloroplast genome database for *Angelica* and has provided valuable insights into the evolutionary relationships among various A*ngelica* species.

## Supplementary Material

Supplemental Material

Supplemental Material

## Data Availability

The genome sequence data that support the findings of this study are openly available in GenBank of the NCBI at (https://www.ncbi.nlm.nih.gov/) under accession no. OQ773546. The associated BioProject, SRA, and Bio-Sample numbers are PRJNA973193, SRR24630317, and SAMN35124822, respectively.
