# Supplementary material for: Complete chloroplast genome of Angelica hirsutiflora Liu et al. 1961 (Apiaceae)
Source: Mitochondrial DNA B Resour. 2024 Apr 5;9(4):470–4. doi: 10.1080/23802359.2024.2335992 (PMC11000599; doi:10.1080/23802359.2024.2335992)
Supplement: Supplemental Material [file TMDN_A_2335992_SM9309.pdf]

Editing Certificate

This document certifies that the manuscript

Complete chloroplast genome and phylogenetic analysis of Angelica hirsutiflora (Apiaceae)

prepared by the authors

Chi-Chun Huang, Tsai-Wen Hsu, Kuo-Hsiang Hung, Wei-Kuang Wang

was edited for proper English language, grammar, punctuation, spelling, and overall style by one or more of the highly qualified native English speaking editors at AJE.

This certificate was issued on **July 23, 2023** and may be verified on the AJE website using the verification code **EAF1-45BA-BEB9-A904-F732** .

Neither the research content nor the authors' intentions were altered in any way during the editing process. Documents receiving this certification should be English-ready for publication; however, the author has the ability to accept or reject our suggestions and changes. To verify the final AJE edited version, please visit our verification page at aje.com/certificate. If you have any questions or concerns about this edited document, please contact AJE at support@aje.com.

AJE provides a range of editing, translation, and manuscript services for researchers and publishers around the world. For more information about our company, services, and partner discounts, please visit aje.com.
