# Supplementary material for: Complete chloroplast genome of Angelica hirsutiflora Liu et al. 1961 (Apiaceae)
Source: Mitochondrial DNA B Resour. 2024 Apr 5;9(4):470–4. doi: 10.1080/23802359.2024.2335992 (PMC11000599; doi:10.1080/23802359.2024.2335992)

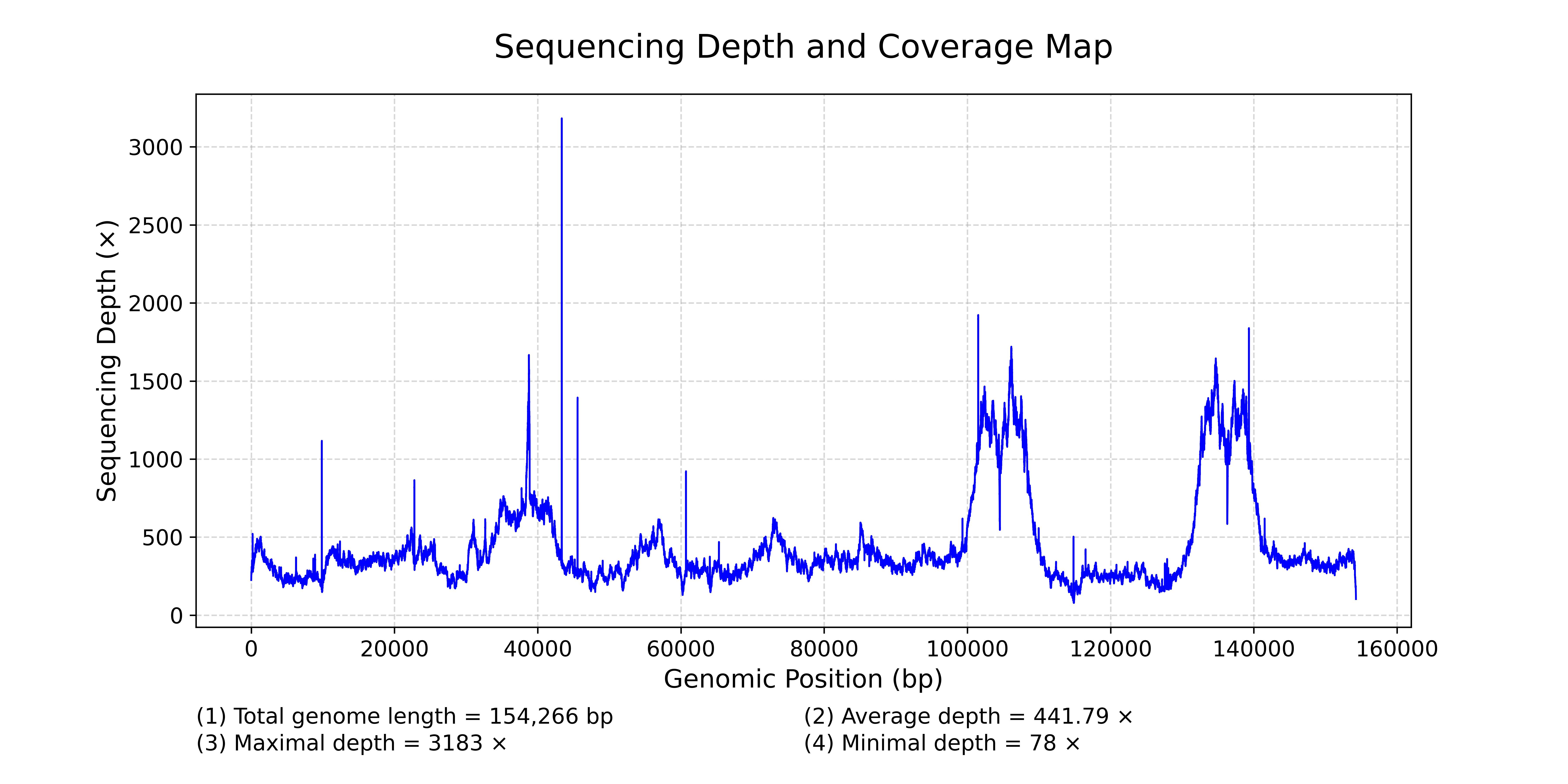


Supplementary Figure 1. Sequencing depth and coverage map of chloroplast genome of *Angelica hirsutiflora*. The horizontal coordinate represents genome position and the vertical coordinate represents the sequencing depth corresponding to that base.

Supplementary Figure 2. (A) Schematic map of the cis-splicing genes of chloroplast genome of *Angelica hirsutiflora*. The direction of the gene is represented by an arrow. Numbers below indicate nucleotide positions of exon and intron. (B) Schematic map of the trans-splicing gene rps12 in the chloroplast genome.


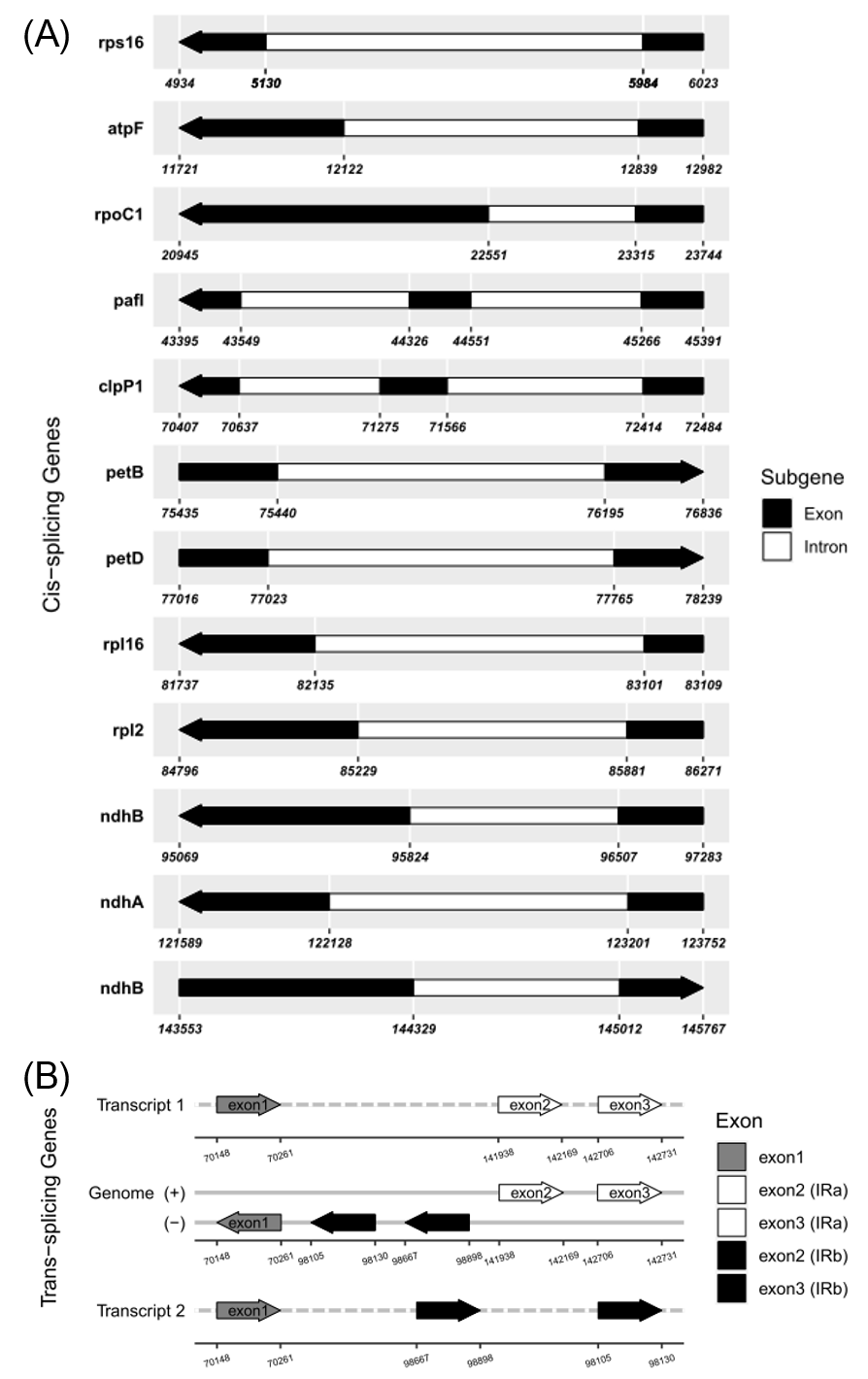

Supplement: Supplemental Material [file TMDN_A_2335992_SM9308.docx]
